# OrthoDB v9.1: cataloging evolutionary and functional annotations for animal, fungal, plant, archaeal, bacterial and viral orthologs

**DOI:** 10.1093/nar/gkw1119

**Published:** 2016-11-29

**Authors:** Evgeny M. Zdobnov, Fredrik Tegenfeldt, Dmitry Kuznetsov, Robert M. Waterhouse, Felipe A. Simão, Panagiotis Ioannidis, Mathieu Seppey, Alexis Loetscher, Evgenia V. Kriventseva

**Affiliations:** Department of Genetic Medicine and Development, University of Geneva Medical School, rue Michel-Servet 1, 1211 Geneva, Switzerland, and Swiss Institute of Bioinformatics, rue Michel-Servet 1, 1211 Geneva, Switzerland

## Abstract

OrthoDB is a comprehensive catalog of orthologs, genes inherited by extant species from a single gene in their last common ancestor. In 2016 OrthoDB reached its 9th release, growing to over 22 million genes from over 5000 species, now adding plants, archaea and viruses. In this update we focused on usability of this fast-growing wealth of data: updating the user and programmatic interfaces to browse and query the data, and further enhancing the already extensive integration of available gene functional annotations. Collating functional annotations from over 100 resources, and enabled us to propose descriptive titles for 87% of ortholog groups. Additionally, OrthoDB continues to provide computed evolutionary annotations and to allow user queries by sequence homology. The OrthoDB resource now enables users to generate publication-quality comparative genomics charts, as well as to upload, analyze and interactively explore their own private data. OrthoDB is available from http://orthodb.org.

## INTRODUCTION

Hypothesizing on gene functions is instrumental for many studies in molecular biology. The most precise functional inferences rely on the concept of orthology, i.e. inheritance of genes by speciation from a common ancestor (1) and thus most likely being ‘equivalent’ genes among species (2). Orthology is also the cornerstone of comparative evolutionary studies. Despite the wide demand, inference of gene orthology across many organisms remains a challenging issue that requires both substantial computational resources and specific expertise, which justifies the creation and maintenance of orthology databases, starting from Clusters of Orthologous Groups (3) and growing to the Quest for Orthologs consortium (4), disseminating expert results to much wider research communities. Each phylogenetic clade or subclade of species has a distinct common ancestor, making the concept of orthology inherently hierarchical. From its conception, OrthoDB explicitly addressed this hierarchy by delineating orthologs at each major species radiation of the species phylogeny (5). OrthoDB data are central for evolutionary studies in many international consortia for genome analyses, particularly in the field of arthropod genomics, e.g. (6–10). Such an exposure to expert scrutiny has earned the OrthoDB methodology a respected reputation and a sizable user base. Our focus consequently was on increasing the coverage of the available species and improving the accuracy of the underlying methodology. The OrthoDB resource is now among the top resources worldwide (11). As the generation of sequencing data grows much faster than experimental interrogations of gene functions, orthology is the best way to link the knowledge acquired in model organisms to a much wider scope of genomics (2). The demand for high-quality orthology predictions is only expected to grow in years to come.

**Figure 1.**
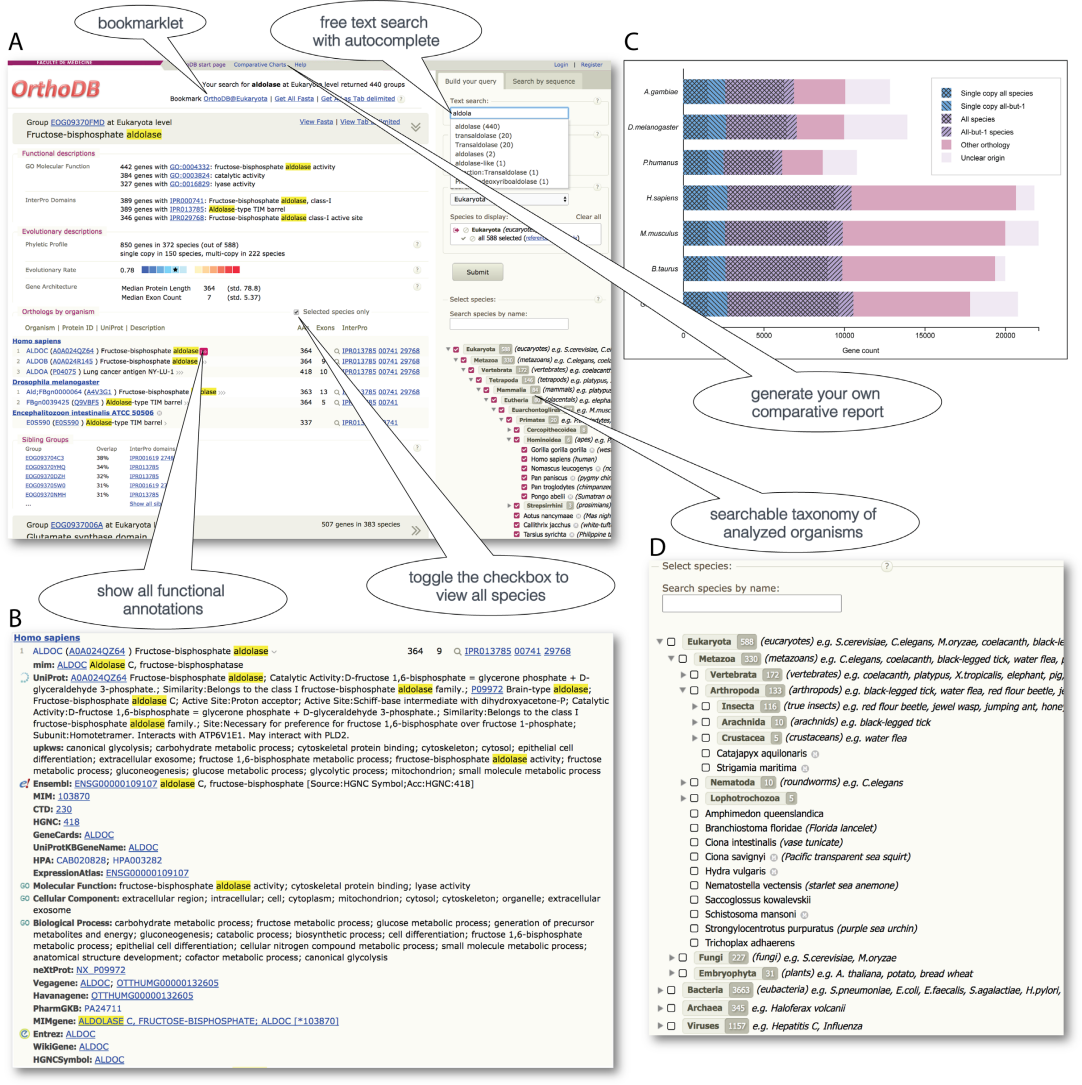
(**A**) An example of the OrthoDB results page for the query ‘aldolase’, showing the ortholog group functional and evolutionary annotations, as well as orthologs from human, fruit fly and a microsporidian parasite. (**B**) An example of all annotations consolidated for the human *ALDOC* gene. (**C**) An example of a comparative report chart that users can generate themselves. (**D**) The detailed organism coverage statistics is shown in the searchable taxonomy.

In this update publication we present OrthoDB v9.1 (http://orthodb.org/v9.1/) that increases the coverage of sequenced species, surpassing any other orthology resource especially in the coverage of eukaryotes (Figure 1D), and further expands the scope and the depth of gathered and synthesized annotations (Figure 1A and B). The web resource presenting the OrthoDB data now enables identified user sessions to analyze custom data sets in the context of the available orthology data, as well as to generate publication quality comparative genomics reports.

## COVERAGE OF ORGANISMS

OrthoDB v9.1 includes a total of 5756 species, providing ortholog groups for the clades of: 3663 bacteria (+28% compared to OrthoDB v8), 330 metazoans (+47%), 227 fungi, as well as adding the clades of 31 plants, 345 archaea and 1157 viruses. Among the metazoans, there are now 172 vertebrates (+64%) and 133 arthropods (+34%). There are 290 orthology levels, that were retrieved from the NCBI Taxonomy (12): 116 eukaryotes (one for plants, 54 for animals, 60 for fungi), 136 for bacteria, 22 for archaea and 16 for viruses.

Protein-coding gene translations were retrieved for vertebrates and plants from Ensembl (13), for arthropods from AgripestBase, AphidBase (14), BeetleBase (15), DiamondBackMoth-DB (16), FlyBase (17), Hymenoptera Genome Database (18), NCBI (19), SilkDB (20), VectorBase (21), wFleaBase (22), as well as the *i*5K pilot project (23) and several other genome consortia. Gene sets for the additional metazoan species were retrieved from the Joint Genome Institute (24). The fungal and viral gene sets were sourced from UniProt (25). We retrieved bacterial and archaeal genomes from Ensembl Bacteria (26), and selected 3663 bacteria and 345 archaea for orthology analysis that have the most complete annotations, as estimated by the proxy of having the most of complete universal single-copy genes (27,28), and that best sample the genetic diversity to ensure the maximum number of clades are represented and to reduce oversampling of certain clades. In the case of strains of the same species the gene set with the highest number of unique genes was kept for orthology analysis.

## THE ALGORITHM AND SOFTWARE

The OrthoDB algorithm for delineation of orthologs is based on Smith–Waterman assessments of gene homology and their subsequent clustering, as has been earlier described (11). Our software is freely available from http://www.orthodb.org/?page=software.

## ORTHODB GRAPHICAL USER INTERFACE

We re-implemented the OrthoDB web interface to be sustainable with the data growth, while maintaining the original website organization (Figure 1A). The ‘*Text search*’ supports querying with various identifiers of proteins, genes, InterPro domains, gene ontology (GO) terms, UniProt etc., as well as gene names, synonyms and functional terms or phrases (placed in quotes for exact phrase matching). The text searches also allow the use of logical operator syntax to build complex queries, e.g. to include variations of a term or to exclude terms. We have also added autocomplete functionality. The ‘*Phyloprofile*’ options allow users to filter the retrieved groups of orthologs by their universality, i.e. presence in most species, and/or the proportion of single-copy orthologs. Despite the dramatic growth of the data volume we still allow users to ‘*Search by sequence*’ against all the protein sequences cataloged in OrthoDB. The level of orthology can be selected from the available radiation nodes. It is worth noting that the results will contain broader groups of genes when a more ancestral radiation level, i.e. a last common ancestor (LCA) closer to the root, is selected, and narrower groups of genes for more closely related species. To enable the most precise comparative studies, OrthoDB has always promoted this concept of hierarchical ortholog groups by computing orthology for different phylogeny radiations. Since the fast growing representation of organisms complicates their practical handling in terms of both selecting and viewing many species at once, we have introduced a ‘*species search*’ of the available organisms, with an autocomplete function and the automatic selection of the LCA of the selected species. That is, when a user selects a set of species of interest, the retrieved groups of orthologs will automatically be selected from the species radiation that represents their LCA. In addition, the results will by default show genes only from these selected species, even though the groups of orthologs were calculated with all species available in OrthoDB for each radiation node. The user then may easily toggle a check box to show, or to hide, the genes from the other species. When a radiation node is selected from the available taxonomy it is interpreted as selecting all of the descending species. As this can result in a large list of genes from many species, the user may choose to view only the ‘reference species’ in order to focus on the best-studied species for which more and better quality annotations are available, with the option to easily toggle the check box to view all species. Users may bookmark their favorite orthology level for quick and easy future searches simply by dragging the bookmark link (e.g. ‘OrthoDB@Insecta’) from the top of the results page into their browser's bookmarks bar. While browsing any website, e.g. a journal article, the user will be able to highlight any text of interest, e.g. a gene name or identifier, and by simply clicking the bookmarklet in their bookmarks bar, the highlighted text will be used to search OrthoDB at the user's favorite orthology level. As in the previous OrthoDB releases, the results can be printed or saved as tab-delimited text, or the protein sequences can be saved in FASTA format. Users looking for larger-scale computational data querying and processing should refer to the OrthoDB application programming interface (API) (see Data Access).

## FUNCTIONAL AND EVOLUTIONARY ANNOTATIONS

Functional annotations available for genes assigned to ortholog groups are arguably the most sought-after information, as they allow for the generation of hypotheses about the inheritance of these functions among the orthologs. In this release we paid particular attention to further enhancing such annotations in terms of both quality and quantity. OrthoDB presents annotations for genes (Figure 1B) as well as for ortholog groups (Figure 1A), i.e. the inferred canonical ancestral gene of each orthology-level LCA. Gene-level annotation records are non-redundant compilations of gene descriptions imported from publicly available resources, which are always back-referenced from OrthoDB by their original identifiers. The major sources of annotations in OrthoDB are from gene records in Ensembl (72.8%), UniProt (72.1%) and NCBI (10.4%), as well as from InterPro (56.2%) and the GO (46.9%), leaving just 11.9% without any mapped functional annotations. We also explicitly present more detailed annotation records for important model organisms such as: *Caenorhabditis elegans* from WormBase (29), *Danio rerio* from the Zebrafish Model Organism Database (30), *Drosophila melanogaster* from FlyBase (17), *Mus musculus* from the Mouse Genome Database (31) and *Saccharomyces cerevisiae* from the Saccharomyces Genome Database (32). In addition, the Database of Essential Genes (33) was used to annotate 207 267 essential genes from 8 model organisms.

Collecting and collating all the available functional annotations from the major resources presents considerable challenges, especially when attempting to focus on the best quality and most useful information. This involved selection of the most pertinent gene annotations by means of programmatic access to Ensembl MySQL (http://ensemblgenomes.org/info/access/mysql) and UniProt SPARQL (http://sparql.uniprot.org/) services, and from NCBI by FTP downloads. All data were processed and consolidated into one-line description per-gene annotation records, these are further click-expandable on the web interface to immediately access the complete record. The relative amount of available annotations per gene is indicated by the size of the click-expandable widget (1–5 chevrons). Annotation of genes is complicated and the sourced data may contain errors. Even though OrthoDB's presentation of the data makes them apparent, users should consider particularly discordant annotations with caution.

We compiled one-line descriptors for 87% of OrthoDB ortholog groups by aggregating all available gene-level functional annotations into ortholog group-level annotations, aiming to provide the user with an overview of the possible functions of the member orthologs at a glance. The compilation of descriptors to briefly but precisely outline functional knowledge in a human-readable language is a non-trivial task. We achieved this by identifying the best scoring single phrase found in any part of the available annotations for all genes in an ortholog group. For each group, all these phrases were matched against the whole body of all annotation records of all genes using a free-text search engine. This body was additionally partitioned according to data origin (UniProt, Ensembl, NCBI, Interpro, GO and UniProt keywords) and the best phrase was evaluated for each partition. The resulting six phrases were ranked using their full-text matching score multiplied by a weight factor empirically evaluated for each partition to impose our preference. Finally, the top ranked phrase was chosen as the representative title of the group. We also annotated 62% of OrthoDB groups with GO and InterPro terms propagating only consistent gene-level annotations.

Evolutionary annotations were computed for each ortholog group from the available genomics data and sequence alignment statistics. These intuitive metrics include: ‘phyletic profile’ that reflects gene universality, i.e. proportion of species with at least one ortholog in a particular ortholog group, ‘duplicability’ that reflects the proportion of multi-copy versus single-copy orthologs in an ortholog group, ‘evolutionary rate’ that reflects the relative conservation or divergence of protein sequence, ‘gene architecture’ that reflects the observed variations of the protein lengths and exon counts of the member orthologs, and ‘sibling groups’ that reflects the sequence non-uniqueness by the fraction of InterPro domains shared with other groups of orthologs. These evolutionary annotations remain a unique feature of OrthoDB.

## MAPPED SPECIES

The completeness of genome assemblies and the quality of their predicted gene models can affect orthology delineation (29). Nevertheless, even incomplete genomes and transcriptomes require comparative interpretation through tentative orthology assignments to make the best use of inferences from better-studied organisms. One approach to this problem is to define ortholog groups with the most complete and best-annotated species and then map genes from species with lower-quality genomic resources onto the core set of ortholog groups from a relevant orthology-level. This two-stage approach also allows for newly-sequenced and annotated genomes to be immediately added to OrthoDB, without waiting for the complete re-build of all ortholog groups. We have thus introduced ‘mapped’ proteomes, representing 22% of the Metazoan species in OrthoDB v9.1 (and none of the other clades), that are clearly identified as such with the ‘M’ symbol in all results tables. Mapping requires that all genes (from the species to be mapped) are first assessed for their homology to all genes from those species included in the high-quality complete clustering set. The same clustering algorithm as for building the core ortholog groups is then applied but now only allowing for new genes to join existing ortholog groups. In this release, to reduce the computational overhead of orthology analysis at the Eukaryota level we selected a representative high-quality subset of 90 species sampling from metazoans, fungi and plants for complete clustering, and subsequently applied the mapping procedure to the remaining 498 eukaryotes.

## BUSCO v2

We previously showed that a substantial fraction of genes is universally present over rather broadly defined clades of organisms and most of these genes are under selection for being maintained as single-copy orthologs (34). While allowing for rare gene duplications or losses, this establishes an evolutionarily-informed expectation that such genes should be found as single-copy genes in any newly-sequenced genome. Hence, we implemented a procedure using Benchmarking Universal Single-Copy Orthologs, called BUSCO (27), to quantitatively measure the completeness of genome assemblies, annotated gene sets and transcriptomes in terms of expected gene content, initially based on OrthoDB v7 data. We employed BUSCO assessments to identify gene sets of generally poorer quality that were excluded from complete orthology clustering, and then subsequently mapped to ortholog groups as described above. Taking advantage of the much more comprehensive species coverage in OrthoDB v9, we developed BUSCO v2 (http://busco.ezlab.org/v2) that includes many more assessment sets for each of the major lineages representing clades with numerous sequenced species. BUSCO v2 also implements improvements to the underlying analysis software, which is now publicly accessible as a GitLab project. Moreover, BUSCO v2 is now available as a virtual machine and can be easily run on any operating system. In addition to assessing completeness, the identified conserved orthologs are ideal candidates for large-scale phylogenomics studies, and the annotated gene models built during genome assessments provide a comprehensive gene predictor training set for use as part of genome annotation pipelines.

## IDENTIFIED USER SESSIONS

In addition to anonymous access to the OrthoDB.org resource we now allow identified user sessions. User identification supports authentication with Facebook or Google credentials, and at no time is any sensitive information passed through our servers as login, registration and password recovery procedures are handled by an established service provider.

Identified users may upload their own data, i.e. FASTA-formatted protein sequences from genes of newly-sequenced genomes. The uploaded gene sets can then be mapped online though a queuing system to the current OrthoDB data at the user specified orthology level. Note that due to practical limitations we restrict the number of species to be used for mapping to fewer than 10, and we leave the choice of these species to the user. The genes mapped to the orthologous groups can be subsequently explored though the website. We will further expand the service to allow BUSCO assessments and tentative gene to GO mapping, taking advantage of the more robust ortholog group-level consensus GO functional annotations. The user-submitted data and analyses will remain private (requiring login) for a limited period of time and then be deleted, unless the user opts for making the data public, in which case these data will become part of incremental OrthoDB updates (subject to passing quality control checks).

## COMPARATIVE GENOMICS REPORTS

Almost all manuscripts presenting newly sequenced genomes include a comparative overview of their gene content showing the total gene count, the fraction of common genes, and the fraction of the single-copy genes. To simplify the drawing of such charts and avoid the common pitfalls, we implemented an online application that uses OrthoDB data to produce user-tailored publication quality vector graphics. More importantly, the fractions displayed on the charts are hyperlinked to easily retrieve the corresponding lists of genes (e.g. all universal single-copy genes from the selected species) for downstream studies. The user interface for generating such comparative genomics charts allows for the selection of up to 20 species available at OrthoDB (Figure 1C), including the privately mapped species.

## DATA ACCESS

We wish to make a particular note to our users wishing to retrieve substantial subsets of data to explore the OrthoDB API. It is documented at http://www.orthodb.org/?page=api and it will return easier to handle data in JSON format, except of course for requests for FASTA or TAB formats. As for the previous versions of OrthoDB, we also provide the data files for bulk download (http://www.orthodb.org/?page=filelist). Users can also navigate to OrthoDB records by following links from FlyBase ‘Orthologs’ section, UniProt ‘Phylogenomic databases’ section or NCBI ‘Additional links/ Gene LinkOut’ section.

## CONCLUSIONS AND PERSPECTIVES

The rapidly growing number of sequenced genomes increases the power of comparative analyses, but also brings new challenges for the scalability of methods and the data presentation to end-users. OrthoDB will continue to provide comprehensive coverage of publicly available annotated genomes and to refine the accuracy of ortholog delineations.
